# Where to from here? A quality improvement project investigating burns treatment and rehabilitation practices in India

**DOI:** 10.1186/s13104-018-3314-9

**Published:** 2018-04-03

**Authors:** J. Jagnoor, C. Lukaszyk, A. Christou, T. Potokar, S. Chamania, R. Ivers

**Affiliations:** 1 The George Institute for Global Health, University of New South Wales, Level 5, 1 King Street Newtown, Sydney, 2042 Australia; 2 Sydney Medical School, University of Sydney, Sydney, Australia; 3 Human and Health Sciences Central, Swansea University, Swansea, Wales UK; 4 Choithram Hospital and Research Centre, Indore, India; 5 Southgate Institute, Flinders University, Adelaide, Australia; 6 Sydney School of Public Health, The University of Sydney, Sydney, Australia

**Keywords:** Burns care, Burns rehabilitation, India, Health services, Health systems, Health professionals

## Abstract

**Objective:**

To describe the capacity of the Indian healthcare system in providing appropriate and effective burns treatment and rehabilitation services.

**Results:**

Health professionals involved in burns treatment or rehabilitation at seven hospitals from four states in India were invited to participate in consultative meetings. Existing treatment and rehabilitation strategies, barriers and enablers to patient flow across the continuum of care and details on inpatient and outpatient rehabilitation were discussed during the meetings. Seventeen health professionals from various clinical backgrounds were involved in the consultation process. Key themes highlighted (a) a lack of awareness on burn first aid at the community level, (b) a lack of human resource to treat burn injuries in hospital settings, (c) a gap in burn care training for medical staff, (d) poor hospital infrastructure and (e) a variation in treatment practices and rehabilitation services available between hospitals. A number of opportunities exist to improve burns treatment and rehabilitation in India. Improvements would most effectively be achieved through promoting multidisciplinary care across a number of facilities and service providers. Further research is required to develop context-specific burn care models, determining how these can be integrated into the Indian healthcare system.

## Introduction

Approximately seven million people sustain burns every year in India [1] with over 61,000 deaths attributed to exposure to fire, heat or hot substances in 2015 [2]. Ten percent of hospitalised burns cases require prolonged hospital treatment lasting over 3 months, with half of surviving patients acquiring permanent disability [3]. The risk factors associated with burns are well known and within India, these include a scarcity of safe fuels, high population density, and additional risk factors associated with intentional burns [4].

Burn care is often poorly organised and under-resourced in low- and middle-income countries (LMICs), exacerbated by the already inadequate health care facilities and fragmented health systems that exist in many of these settings [5]. Potentially damaging local traditional first aid practices provided to patients prior to attaining definitive medical care, such as applying urine, mud, or cow dung directly to a burn wound, may compound burn care further [6]. Burns have the highest average direct medical costs of all injury types in LMICs [7] due to lengthy periods of hospitalization, operative costs, and costs associated with diagnostic tests, dressings, medications and need for long rehabilitation [8–10]. In many LMICs, a large proportion of these costs are borne by the patient. In Vietnam, the average out-of-pocket cost per burn injury hospitalization is over US$270 [10]. It is therefore important to understand the context of available resources and local health system structure when making recommendations for the management and delivery of burns care services.

The aim of this project was to document current practices for burns care in India, particularly for care after the acute phase of injury and for rehabilitation services provided post-discharge. This information was used to inform further research questions and guided the development of topic guides for additional large-scale qualitative work.

## Main text

### Methods

This project was initiated in 2015 as part of a broader suit of work investigating the availability, acceptability and effectiveness of services providing burns care in India. Seven secondary and tertiary medical facilities in Tier-II and Tier-III metropolitan cities across four states in India were selected for inclusion in the study (Table 1). Purposive sampling was used to identify government and non-government hospital facilities. Facility representatives were approached by research personnel and invited to participate in the project, with written approval provided by facility Directors from all hospitals approached following the review of study protocol and topic guides. Health professionals involved in providing burns care were briefed on project aims and the purpose of the research, given an opportunity to ask questions about the project both to the project lead and to management prior to their participation. Consent was implied by participation in the study, with participation perceived to be low risk by heads of medical facility. The assurances of confidentiality were provided to stakeholders, including agreement that the names of individuals and organisations would not be included in any outputs generated. It was unfeasible to include rural and district level health services as burns care is extremely limited in these settings. Health professionals were requested to participate in one-to-one or group based consultative meetings led by the first author (JJ) in October and November 2015. The first author is a public health systems researcher who is independent of each medical facility, promoting open dialogue during meetings and ensuring participation would have no direct impact on the employment or clinical duties of health professionals. Health professionals were aware that their experiences and suggestions would be included in a report prepared for the World Health Organisation and in other associated academic publications. A topic guide was used by the facilitator to ensure all major points of enquiry were addressed during the meetings (Table 2). An attempt to meet with all levels of clinical staff involved in burn care was made, allowing for a diverse range of perspectives to be documented. Consultative meetings were held until no new themes arose from discussions, indicating that data saturation had been reached. Notes taken during the meetings were thematically analysed using a Grounded Theory approach to identify the key components, strengths and shortfalls of existing burns treatment and rehabilitation strategies. No statistical analysis was performed due to the qualitative nature of the data collected for this project. Co-authors discussed the output until consensus was reached upon the major themes identified by health professionals.

**Table 1:** Hospital sites selected for stakeholder consultations and characteristics of health professionals involved

Site	Capacity	Patient flow	Resources with burn care and rehabilitation team	Health professionals involved in consultations
Site A, Public Hospital, New Delhi	65 bedded burns unit	5800 patients admitted each year, with 55% surviving and nearly 100% loss to follow up post 3 weeks of discharge, with population base of up-to 500 km	Team of 20 consultants (including residents), 15 nurses, 2 physio-therapists, 7 ward attendants	Professor; head of the department, male Senior resident, female Physiotherapist, female
Site B, Public Hospital, New Delhi	50 bedded burns unit	4300 patients admitted each year, with nearly 50% surviving. Loss to follow up is high and population base of up-to 500 km	Team of 17 consultants, 12 nurses, 5 ward attendants, 1 dietician. Physiotherapists, psychiatrist and social workers are consulted at on call basis	Professor; head of the department, male Psychiatrist, female
Site C, Private Hospital, Gujarat	4 bedded unit	12–16 patients admitted each year. 90% surviving all patients followed up till best possible rehabilitation	Team of 2 consultants, 4 nurses, 2 ward attendants, 1 physiotherapist, 1 dietician and an on-call social worker	Lead clinician, male Physiotherapist, male
Site D, Public Hospital and educational institute, Gujarat	24 bedded burns unit and 7 beds in general surgery unit	4800 patients admitted, numbers for OPD are uncertain as burns patients are looked at general surgery	Team of 7 consultants and 15 nursing staff, ward attendants 3. Physiotherapy department consulted on need basis	Senior resident, general surgery, male Senior resident, burns, female Head of physiotherapy department, male
Site E, Private Hospital, Mumbai	10 bedded unit	300 admission each year. No regular OPD	Team of 4 consultants, 25 nurses and brothers, 1 physiotherapist, 1 psychologist, 1 dietician, 2 social worker	Psychologist, female Social worker, female
Site F, Charitable Hospital, Indore	12 bedded unit	250 admission every year and an OPD of 750 patients	Team of 5 consultant staff, 9 nurses, 7 paramedical staff, 1 dietician, 1 physiotherapist, 1 psychologist, 1 occupational therapist	Burns surgeon, female General surgeon, female Psychologist, female Occupational therapist, male Physiotherapist, female
Site G, Public Hospital, Mumbai	8 bedded burns unit, and 4 beds in general surgery unit	700 admitted patients, usual practice is for male patients to be sent to general surgery and OPD of 1000 patients	Team of 3 doctors, 3 nurses, 1 physiotherapist, 1 social worker, 2 occupational therapists and 1 dietician are consulted on referral basis	Small group discussion with the burns treatment team

**Table 2:** Topic guide used for health professional consultation

1.	Does your facility/professional group have an overarching rehabilitation plan/strategy? If yes, please briefly describe
2.	How is it determined in which practice setting (inpatient, outpatient, at home) a patient will receive rehabilitation care? e.g. admission criteria
3.	What objective assessment of rehabilitation potential occurs before a patient is accepted into the service? e.g. physician assessment, use of tools like FIM, therapist assessment
4.	What are the enablers and/or barriers associated with patient flow across the continuum of care? What are the enablers and/or barriers specifically associated with rehabilitation care at admission, whilst in hospital, or post discharge? e.g. workforce shortages, defined clinical pathways, patient resource limitations
5.	Are there objective measures regarding how much rehabilitation a patient should receive, or when to stop providing care? e.g. discharge criteria, defined funding eligibility limits
6.	Approximately what proportion of the health system’s rehabilitation care occurs in inpatient vs. outpatient vs. at home?
7.	Who makes up the rehabilitation team at your hospital? e.g. dietician, physiotherapist, psychologist
8.	Are there usual patterns of treatment frequency, intensity, and duration? If so, can you describe these? e.g. a patient in inpatient rehabilitation would be seen twice daily for an hour each until able to be seen in outpatient
9.	How does a patient move from one practice setting to another, for example from inpatient to outpatient? Is this based on functional measures, based on funding or based on access?
10.	What are the key rehabilitation issues you identify in burns survivors? This can range from prevention and first aid through to rehabilitation

### Results

A total of 17 health professionals were involved in the consultation process including nurses, allied health professionals such as residents, consultants, physiotherapists, social workers, dietician, plastic surgeons and general surgeons (Table 1).

The care provided to burns patients by hospitals was heterogeneous across settings (Table 1). As care varied across sites, the experiences and challenges faced by health professionals were also diverse. Illustrative quotes supporting each of the themes below are presented in Table 3.

**Table 3:** Key themes identified by health professionals with illustrative quotes

Theme	Quote
*Lack of awareness of first aid burn care among community members*	“*But madam, we have to provide First Aid, patients can’t do it for themselves*.” [Nurse, Public Hospital] “*Lack of awareness is a big issue. Appropriate first aid can go a long way.”* [Resident, Public Hospital] *“You would be surprised that even doctors do not know what to do in case of chemical burns, not water alkali*- *right! There is need for IEC material (information, education and communication materials) both for public and health professionals.”* [General Surgeon, Charitable Hospital]
*Human resource, training and stigma*	*“Man power is our biggest challenge.”* [Consultant, Public Hospital] *“Plastic surgeons don’t want to attend to burns patients. We as general surgeons do a range of surgeries, we can manage till wound healing. However after that I do not feel confident for splinting*- *that I am providing the best treatment to these patients.”* [General Surgeon, Public Hospital]
*Infrastructure and resources*	*“Dressing is an issue, it is very expensive. We use banana leaves, there is clinical evidence* - *you can see publication on this by Dr Gore.”* [Senior Resident, Public Hospital] *“Stretchers, sterilization and mobility can be challenging.”* [Physiotherapist, Public Hospital] *“We run a skin bank. As you can see here it is not cheap*- *the process takes days but prognosis is very good. Our outreach team has to put in a lot of effort to convince families and staff needs to ensure there is no bleeding. It is a very skilful job”.* [Microbiologist, Not-for-profit Hospital]
*Lack of standardization in treatment practices*	*“We don’t have standardised referral procedures. It depends on availability of bed and degree of burns, patient prognosis between emergency departments, burns and general surgery”* [Senior resident, Public Hospital]
*Lack of guidelines for rehabilitation*	“*There was this female patient and she was from a good family. She came back with contractures in her hand after 3* *months. She was just lazy. She did not do the exercises we told her*.” [Resident, Public Hospital] “*It is very late in practice after years of experience that physicians begin to recognise the relevance of mental health in patient treatment*.” [Consultant, Charitable Hospital]

#### Lack of awareness of first aid burn care among community members

Practitioners reported that poor patient outcomes were not only related to the severity of the burn injury, but also due to poor awareness of first aid measures required to be carried out immediately after the burn injury, and/or inaccurate assessment of burn severity by health workers in community settings. Physicians reported none of their patients had used running water for 20 min for first aid treatment of a burn. For thermal burns, a range of products including ink, ice, honey, turmeric, mud, egg, ghee (saturated butter) and toothpaste were commonly applied to burn sites by carers.

#### Human resource, training and stigma

Human resource challenges were an issue at all sites. This was not limited to trained health professionals, but extended to cleaners and ward assistants, posing major challenge in burns care where infection control is critical. Whilst most public hospitals reported major challenges in infection control, private and charitable hospitals had dedicated teams for this. Consequently, use of prophylactic antibiotics was common practice in public hospitals, whilst other health facilities with better infection control measures only used antibiotics in the peri-operative phase, or with suspected or established sepsis.

General surgeons acting as primary treating physicians for burns cases reported lack of competence and in managing burns, not well equipped particularly for splinting. Surgeons reported the need for specialized, continued training and education in burns care in order to effectively manage and treat burn injuries.

It was also reported by all health-provider types that working in burns care was not rewarding; recovery outcomes were poor, and the poor post–discharge compliance was discouraging for health professionals. Burns survivors were also severely stigmatised by resident patients in the health facility which consequently affected their access to facilities. Taboo also surrounded the sight of disfigured burns patients; burns survivors were kept out of sight of maternity patients due to prevailing beliefs that the unborn child would carry the same disfigurement/scarred looks.

#### Infrastructure and resources

Barriers to the provision of adequate infection control and management were reported by all sites. A shortage of beds in public hospitals prevented the isolation of burns patients, particularly in larger metropolitan hospitals, increasing the likelihood of spread of infection. Difficulties with accessing medication for pain relief, fluid resuscitation and wound dressing were reported. Some indigenous methods of care were used, including banana leaf dressings that were low cost and locally available. Due to resource limitations, terminal patients that had little hope of survival could often not be admitted for comfort care.

#### Lack of standardization in treatment practices

Practices for the treatment of burns at each hospital varied in regard to the dressing material used, the frequency the dressing was changed, and/or the health professionals involved in the treatment.

Psychologists were generally not included in burn care teams and the physicians interviewed agreed they were often unable to diagnose or assist patients with psychological challenges including post-traumatic stress disorder (PTSD), anxiety and depression. It was acknowledged that addressing psychological issues was particularly important for improved adherence to rehabilitation advice and recovery.

#### Lack of guidelines for rehabilitation

None of the public hospital sites used objective tools for rehabilitation assessments, nor did any site have a planned rehabilitation prescription or structured program. Despite this, staff generally understood the need for treatment and care across the various rehabilitation domains (physical, psychological and social and community) but were limited in their capacity and the resources available to them. Significant follow-up, including vocational re-training, was generally managed by off-site non-government organisations.

Limited rehabilitation was provided for psychosocial support, particularly at public hospitals. Practices were sporadic and dependent on referral from physicians. Dieticians at three hospitals documented treatment and progress by monitoring weight changes and blood reports.

Discharge rehabilitation planning was in place at three hospital sites, however there was significant loss to follow up with only one private hospital having an outreach plan in place. The major concerns of all health professionals were post-discharge compliance with use of pressure garments for hypertrophic scars, and the development of contractures. Plastic surgeons also raised issues related to self-image, stigma and resultant isolation experienced by survivors. Some of the overarching challenges identified with post-discharge rehabilitation included low socio-economic status and education levels, lack of awareness of the importance of rehabilitation, and distance from rehabilitation services. Commute to the health facility for rehabilitation was observed as a major challenge for treatment compliance.

### Discussion

As part of this quality improvement project, a number of barriers were identified for the provision of care for burn injuries at primary, district and tertiary health services in India. This study demonstrates how a lack of operational standards for burns care, the varied knowledge and skills of health professionals in providing burns care, together with resource shortages, greatly impacts the quality of care for burns patients.

The absence of standardised, clinical guidelines for acute burns treatment and rehabilitation were identified as a key issue by a range of health professionals based at primary and district health facilities. Standard setting is a crucial strategy for improving quality in health care, strengthening health systems, and enhancing patient outcomes in a cost-effective manner [11]. Interburns, a charity advocating for better burns care, has developed guidelines with an objective to define operational standards for different levels of burn care service in LMICs [12]. This includes guidelines on the resources and activities necessary to ensure optimal outcomes for patients and a framework for education and training programmes for burn care professionals in the context [13]. Such a guideline can be used to form the basis of developing a standard for burn care in India.

At many sites, the provision of hospital-based rehabilitation services was restricted by resource shortages, with no standardised burns rehabilitation programs offered between facilities. Patients were deterred from returning to district-level facilities for rehabilitation as out-patients due to long travel distances, associated travel costs and stigma associated with the appearance of their burn injury. These issues suggest that developing community-based burn rehabilitation services may be more successful in providing appropriate care in this context. There has been a rise in the adoption of community-based rehabilitation strategies since the initiation of the World Health Organisations’ community-based rehabilitation (CBR) strategy in 1978 [14]. Such CBR strategies enable communities to develop and implement services to meet local needs while promoting the use of local human, financial and material resources where possible to increase the likelihood of sustainability [15].

## Limitations

- Only perspectives from selected health professionals are presented in this manuscript. It would be valuable to investigate experiences reported by burns patients from time of injury to long-term follow-up.
- All medical facilities included in this study are hospitals located in urban areas. It would be beneficial to speak with health care providers working in rural primary and secondary healthcare services to gain a better understanding of burns treatment provided at the community level when tertiary care is either not sought or delayed.
- India is a diverse nation with significant differences in population demographics, population distribution, and the availability and quality of health services between states. This study sampled health professionals from a range of public and private health facilities, however in only 4 out of 29 states, potentially limiting the generalisability of study outcomes for the national level.

## Abbreviations

CBR: community-based rehabilitation
LMIC: low and middle income country
NGO: non-government organisation
PTSD: post-traumatic stress disorder
US: United States
